# Optical Coherence Tomography Angiography Characteristics Serve as Retinal Vein Occlusion Therapeutic Biomarkers for Dexamethasone Intravitreal Implant

**DOI:** 10.1155/2021/3510036

**Published:** 2021-10-13

**Authors:** Ke Shi, Xiaodong Sun, Jingfa Zhang

**Affiliations:** ^1^Department of Ophthalmology, Shanghai General Hospital, Shanghai Jiao Tong University, School of Medicine, Shanghai, China; ^2^National Clinical Research Center for Ophthalmic Diseases, Shanghai, China; ^3^Shanghai Key Laboratory of Fundus Diseases, Shanghai, China; ^4^Shanghai Engineering Center for Visual Science and Photomedicine, Shanghai, China

## Abstract

**Background:**

Retinal vein occlusion (RVO) is the second most common vision-threatening retinal vascular disease. Intravitreal dexamethasone implant has been applied to treat macular edema secondary to RVO (RVO-ME). However, the alteration of morphologic features detected with optical coherence tomography angiography (OCTA) has not been fully studied in RVO-ME patients before and after the treatment.

**Objective:**

This study is aimed at identifying potential therapeutic targets in RVO with integrative bioinformatic analysis and compares the OCTA characteristics alterations in patients with RVO-ME receiving injection of dexamethasone intravitreal implant.

**Methods:**

Bioinformatic analysis was analyzed in GSE101398 dataset from the Gene Expression Omnibus database. Multiple functional enrichment analyses were performed, and protein-protein interaction network was constructed to visualize the key node genes. Eleven eyes with RVO-ME were examined with OCTA before and after intravitreal dexamethasone implant. The OCTA parameters, including macular thickness, vessel density, foveal avascular zone parameters, the number of hyperreflective foci (HRF), area of cystoid edema, and subretinal fluid (SRF), were compared. The correlation was analyzed between best-corrected visual acuity (BCVA) and OCTA parameters.

**Results:**

A total of 79 differentially expressed genes were identified. Functional enrichment analyses revealed the enriched inflammatory events in RVO. In RVO-ME, Pearson correlation revealed that baseline BCVA was positively correlated with the area of SRF and central macular thickness, while no correlation was detected between baseline BCVA and HRF number or the area of cystoid edema. The visual acuity improved, and the central macular thickness was decreased after intravitreal dexamethasone implant injection. Besides, the number of HRF, the area of cystoid edema, and SRF were significantly alleviated after dexamethasone intravitreal injection.

**Conclusion:**

Retinal inflammation plays a crucial role in RVO pathogenesis. The imaging biomarkers of RVO including Müller glial intracellular edema, and retinal pigment epithelium dysfunction, could be assessed in OCTA and attenuated by intravitreal dexamethasone implant effectively.

## 1. Introduction

Retinal vein occlusion (RVO) is a common retinal vascular disorder to cause visual impairment worldwide. RVO can be classified into two types according to the occlusion site, i.e., central retinal vein occlusion (CRVO) and branch retinal vein occlusion (BRVO). Rogers et al. [1] combined fifteen studies including 68,751 individuals and reported that the prevalence of BRVO (4.42 per 1,000) was higher than CRVO (0.80 per 1,000) in all racial populations. To date, no effective treatments are available for the prevention or treatment of RVO. Present treatments are mostly focused on the complications of RVO, such as macular edema (ME) and neovascular glaucoma, with antivascular endothelial growth factor (anti-VEGF) agents or laser photocoagulation.

ME is the predominant cause of vision deterioration in RVO Hayreh [2], distinguished by fluid accumulation in the macular region [3]. The breakdown of blood-retina barriers (BRB) accounts for the main cause of ME formation [4]. Although the pathophysiology of RVO is still not fully understood, vascular endothelial growth factor (VEGF) is widely acknowledged to play a critical role in the pathogenesis of RVO. Anti-VEGF therapy has been shown to be effective to reduce ME and improve visual acuity in most RVO cases. However, monthly anti-VEGF injections laid a burden for most patients; of some, the ME treatment cannot meet the satisfactory effect in terms of visual acuity improvement and ME reduction, indicating other factors besides VEGF might be involved in the pathogenesis of RVO-ME, especially inflammation including inflammatory cells and inflammation-related cytokines. Steroids, the potential anti-inflammatory agent, could decrease the production of several inflammation-related and propermeability proteins, such as VEGF, interleukin 1, intercellular adhesion molecule 1, and monocyte chemoattractant protein-1, as well as maintain the integrity of BRB, thus providing the rationale for steroid-based therapy to treat RVO-ME [5, 6]. The dexamethasone intravitreal implant, sustainedly releasing corticosteroid, has been shown beneficial effect in patients with RVO-ME. However, alteration of morphologic features detected with optical coherence tomography angiography (OCTA), such as hyperreflective foci (HRF), cystoid edema, and subretinal fluid, has not been fully studied in patients with RVO-ME before and after intravitreal dexamethasone implant treatment.

In this study, we utilized the online database and performed bioinformatic analysis to determine the possible inflammation-related factors involved in the pathogenesis of RVO. To verify this, 11 eyes from 11 patients with RVO-ME were employed and observed before and after intravitreal dexamethasone implant treatment, with special focus on the inflammation involvement in the pathogenesis of RVO. The preliminary results showed that inflammation played a crucial role in RVO, and dexamethasone was effective to decrease inflammation in patients with RVO, such as the decreased number of hyperreflective foci (HRF) and subretinal fluid (SRF) and the intracellular edema of Müller glia. The possible mechanism for the pathogenesis of RVO-ME and the effect of dexamethasone on RVO-ME were also proposed in the current study.

## 2. Materials and Methods

### 2.1. Gene Expression Data Source

GSE101398, the gene expression profiling of the laser-induced RVO mice model [7], was derived from the Gene Expression Omnibus (GEO, https://www.ncbi.nlm.nih.gov/geo/), a database repository of high-throughput gene expression profiles. Three laser-induced RVO mice and three controls were included in GSE101398. The platform for GSE101398 was GPL15103 (Illumina HiSeq 1000[Mus musculus]). The *H* (hallmark gene) sets, C2 curated gene sets, and C5 ontology gene sets were downloaded from the Molecular Signatures Database (MSigDB, https://www.gsea-msigdb.org/gsea/msigdb/) [8].

### 2.2. Differentially Expressed Genes (DEGs) Analysis

The limma [9] package in *R* software version 4.0.4 (RRID:SCR_001905, R Foundation for Statistical Computing, Vienna, Austria) was employed to screen the DEGs with the filter of ∣logFC |  (an absolute log2 value in the fold change of the gene expression) > 1.5 and *p* value <0.01. The org.Hs.eg.db *R* package was used to select the common genes shared by both mouse and human. Subsequently, DEGs were visualized in volcano plot and heatmap with ggplot2 *R* package and pheatmap *R* package [10], respectively.

### 2.3. Function Enrichment Analysis

Gene Ontology (GO) [11] function analysis, including biological process (BP), cellular components (CC) and molecular function (MF), and Kyoto Encyclopedia of Genes and Genomes (KEGG) [12] analysis were used to identify the characteristic biological features and pathways involved in the pathogenesis of RVO. The results were visualized by clusterProfiler [13] and ggplot2 *R* package. Terms and pathways that met the criteria of both *q* value <0.01 and *p* value <0.01 were considered significantly enriched.

### 2.4. Gene Set Enrichment Analysis (GSEA) and Gene Set Variation Analysis (GSVA)

*R* package clusterProfiler was utilized to perform GSEA with C2 curated gene sets from MSigDB as the reference. The three thresholds for significantly enriched gene sets were *p* value <0.01, *q* value <0.05, and normalized enrichment score (∣NES | >1.5). The five upregulated and five downregulated pathways with significant enrichment scores were visualized using gseaplot2 and ggridges *R* package.

Moreover, GSVA [14] was performed to determine the difference of enrichment pathways. Hallmark, GO, and curated KEGG gene sets from MSigDB were used as the references. An adjusted *p* value of 0.05 and logFC value of 0.05 were set as the cut-off to identify significant enrichment pathways between normal and RVO samples.

### 2.5. Protein-Protein Interaction (PPI) Analysis

PPI network of the DEGs was performed using the Search Tool for the Retrieval of Interacting Genes (STRING version 11.0b, RRID:SCR_005223, https://string-db.org/) [15] to explore the interaction of DEGs. An interaction with a combined score higher than 0.4, which was a widely used threshold, was considered statistically significant. Ten genes with most nodes were selected and visualized by barplot *R* package.

### 2.6. Patients and Intravitreal Injection of Dexamethasone Intravitreal Implant

The present study was a retrospective cohort study, and 11 eyes of 11 treatment-naïve patients with RVO-ME were included. The study was conducted in accordance with the tenets of the Declaration of Helsinki. All the participants enrolled in this study were seen at Department of Ophthalmology, Shanghai General Hospital affiliated to Shanghai Jiao Tong University from 1 October 2020 to 20 April 2021. All individual participants included in the study signed written informed consent to participate in this study and publish their data and photographs. The clinical research protocol was approved by ethical committee of Shanghai General Hospital affiliated to Shanghai Jiao Tong University (2020KY205).

All individuals have undergone comprehensive ophthalmic examinations at baseline and during the follow-up visit, including best-corrected visual acuity (BCVA), intraocular pressure (IOP), slit-lamp examination, fundus photography, and optical coherence tomography angiography (OCTA) examination with the Angiovue Imaging System (RTVue XR Avanti, Optovue Inc, Fremont, CA, USA).

The inclusion criteria were diagnosis of RVO-ME, which was made based on the clinical findings including the presence of preretinal and intraretinal flame-shaped hemorrhage involving one or more quadrants, dilated and tortuous veins, macular edema, and subretinal fluid. The main exclusion criteria were as follows: (1) previous treatments for RVO, including intravitreal injection of anti-VEGF or corticosteroid drugs and laser photocoagulation; (2) other retinal diseases including age-related macular degeneration, vitreous hemorrhage, diabetic retinopathy, and choroidal neovascularization; and (3) history of intraocular surgery within 3 months, uveitis, pathologic myopia (< −6.0 diopters), corneal, or lens opacites.

All the patients were treated with intravitreal injections of dexamethasone intravitreal implant (0.7 mg, OZURDEX®, Allergan plc, Dublin, Ireland). Intravitreal injection was administrated as previously described [16].

### 2.7. Outcome Assessment

Macular 6 × 6 mm^2^ scan was acquired with OCTA, which was performed one week after dexamethasone implantation in all patients. The following parameters were analyzed by OCTA AngioVue software version 2017.1 (Optovue Inc, Fremont, CA, USA), including central macular thickness and vessel density in the fovea (0-1 mm diameter from central fovea), parafovea (1-3 mm diameter from central fovea), perifovea (3-5 mm diameter from central fovea), and foveal avascular zone (FAZ), as well as the En-face images of the superficial slab, deep slab, outer retina slab, etc. The boundary would be adjusted manually in the case of imprecise layering. FAZ area, perimeter, and foveal vessel density within a 300 *μ*m wide region of FAZ (FD-300) were measured by OCTA AngioVue software. The areas of cystoid edema and SRF were analyzed using ImageJ version 1.46r (RRID:SCR_003070, National Institutes of Health, Bethesda, MD, USA). The pairs of En-face images and angiography images were imported, merged into a single 24-bit RGB color image, and analyzed with the ImageJ software. The number of HRF was manually counted in fovea-spanning horizontal B-scan images and recorded at three slabs: superficial slab, deep slab, and outer retina slab. HRF was defined as the hyperreflective dot less than 30 *μ*m in diameter as reported by Bolz and colleagues [17].

### 2.8. Statistical Analysis

All the statistical analyses were performed using *R* software, EmpowerStats version 2.0 (X&Y solutions Inc., Boston, MA, USA), and SPSS Statistics version 23.0 (RRID:SCR_019096, IBM, Chicago, IL, USA). BCVA was converted to the logarithm of the minimal angle of resolution (logMAR) equivalents for analysis. Two-way analysis of variance (ANOVA) with Tukey's post-hoc test was conducted to compare the number of HRF in different slabs. The paired Student's *t*-test was used to compare differences in other numeric parameters between baseline and post-injection. Pearson correlation was used to assess the correlation between visual acuity and OCTA characteristics. *p* value <0.05 was considered to suggest a significant difference.

## 3. Results

### 3.1. Research Flow Chart

**Figure 1:**
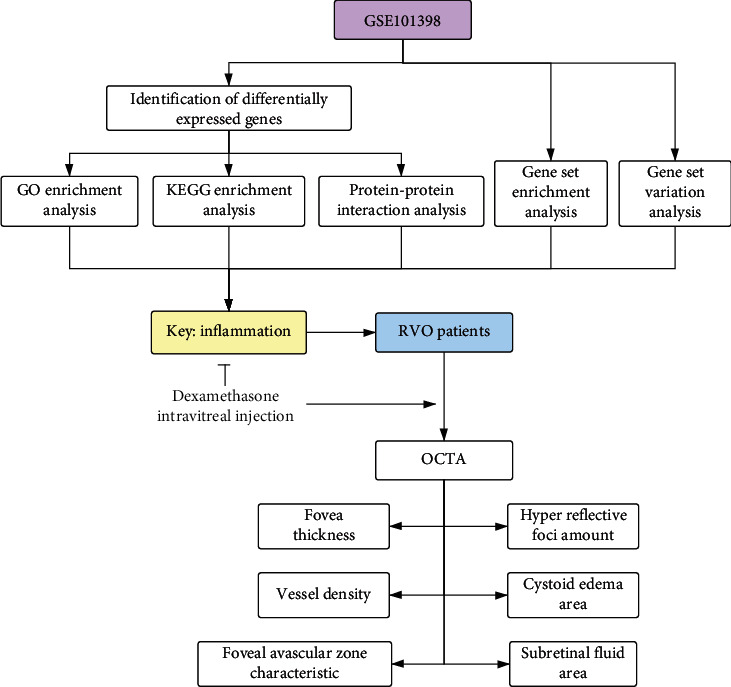
Brief flow chart of this study. GO: Gene Ontology; KEGG: Kyoto Encyclopedia of Genes and Genomes; RVO: retinal vein occlusion; OCTA: optical coherence tomography angiography.

The present study consisted of two parts, i.e., bioinformatic analysis and clinical study. The flow chart was shown in Figure 1. The bioinformatic analysis was performed using GSE101398 dataset. Screening of DEGs was followed with GO and KEGG enrichment analysis, as well as PPI network construction, to explore the significant enrichment pathways and genes involved in RVO, focusing the effect of inflammation on retina. GSEA and GSVA were implemented to detect the significant gene set in RVO. The clinical study was performed in 11 treatment-naïve patients with RVO-ME, who received single intravitreal injection of dexamethasone intravitreal implant. OCTA was applied to obtain various imaging features including central macular thickness, vessel density, FAZ area, HRF, and SRF.

### 3.2. Identification of DEGs in Laser-Induced RVO Mice Model

**Figure 2:**
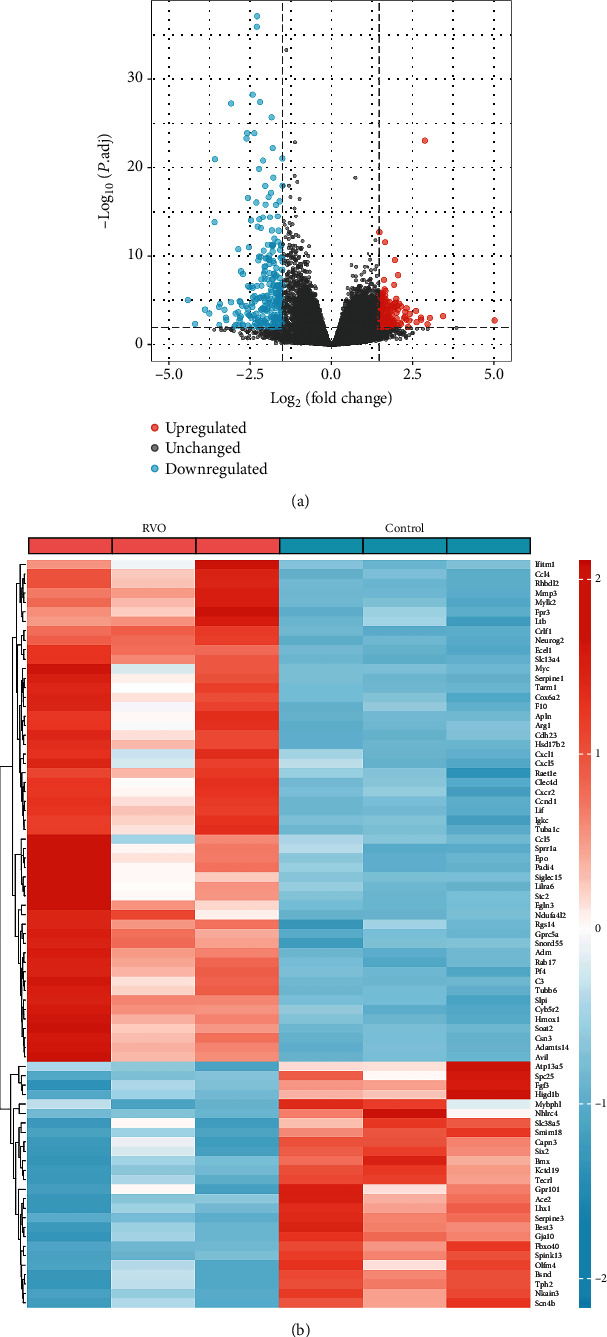
Identification of differentially expressed genes (DEGs) in the RVO mice model. (a) Volcano plot of DEGs extracted from GSE101398 expression matrix. (b) Heatmap of DEGs shared by human and mouse species.

A total of 417 DEGs were extracted from GSE101398 based on the defined criteria (Figure 2(a)). After employing the org.Hs.eg.db *R* package for screening out genes shared by mice and human, 79 DEGs including 53 upregulated genes and 26 downregulated genes were identified (Figure 2(b)).

### 3.3. Function Enrichment Analysis Demonstrated That Inflammation-Related Events Were Involved in RVO

**Figure 3:**
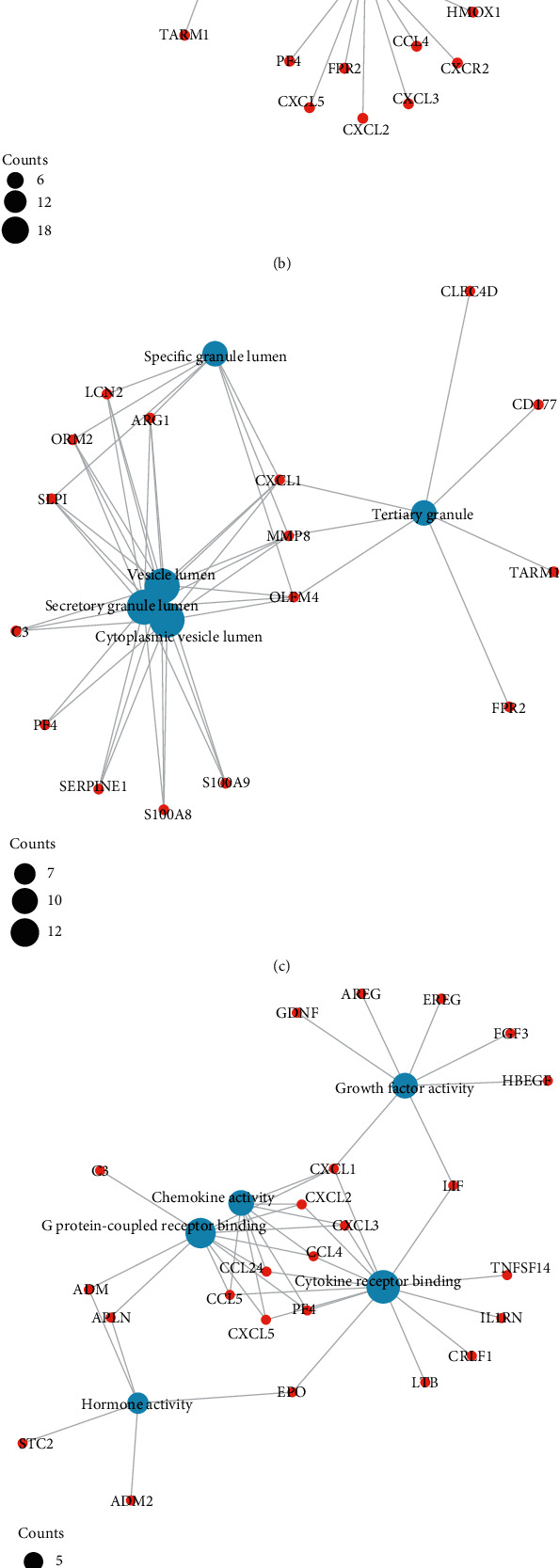
Gene Ontology (GO) and Kyoto Encyclopedia of Genes and Genomes (KEGG) enrichment analysis in RVO. (a) Bubble plot illustrated GO enrichment terms of DEGs in RVO including biological processes (BP), cellular components (CC), and molecular functions (MF). (b)–(d) ClueGo network of DEGs and enriched GO terms of BP (b), CC (c), and MF (d). (e) Bubble plot illustrated enrichment of DEGs in KEGG pathways in RVO. (f) ClueGo network of DEGs and enriched KEGG pathways.

As illustrated in Figures 3(a)–3(d), GO enrichment analysis suggested that for BP, DEGs were significantly enriched in the following processes, e.g., leukocyte migration and cell-cell adhesion, response to steroid hormone, and positive regulation of leukocyte activation and inflammatory response, related to inflammation [18]. For CC, DEGs were significantly enriched in vesicle lumen and granule lumen which were related with cell adhesion and migration [19]. Regarding MF, DEGs were significantly enriched in cytokine receptor binding, chemokine, and growth factor activity that were relevant with inflammation process [20].

The results of KEGG pathway enrichment suggested that DEGs were mostly enriched in nuclear factor kappa B (NF-*κ*B), tumor necrosis factor (TNF), and hypoxia inducible factor 1 (HIF-1) signaling pathways which were largely associated with inflammatory cascades (Figures 3(e) and 3(f)).

### 3.4. GSEA and GSVA Showed Several Pathways Related to Inflammation Were Involved in RVO

**Figure 4:**
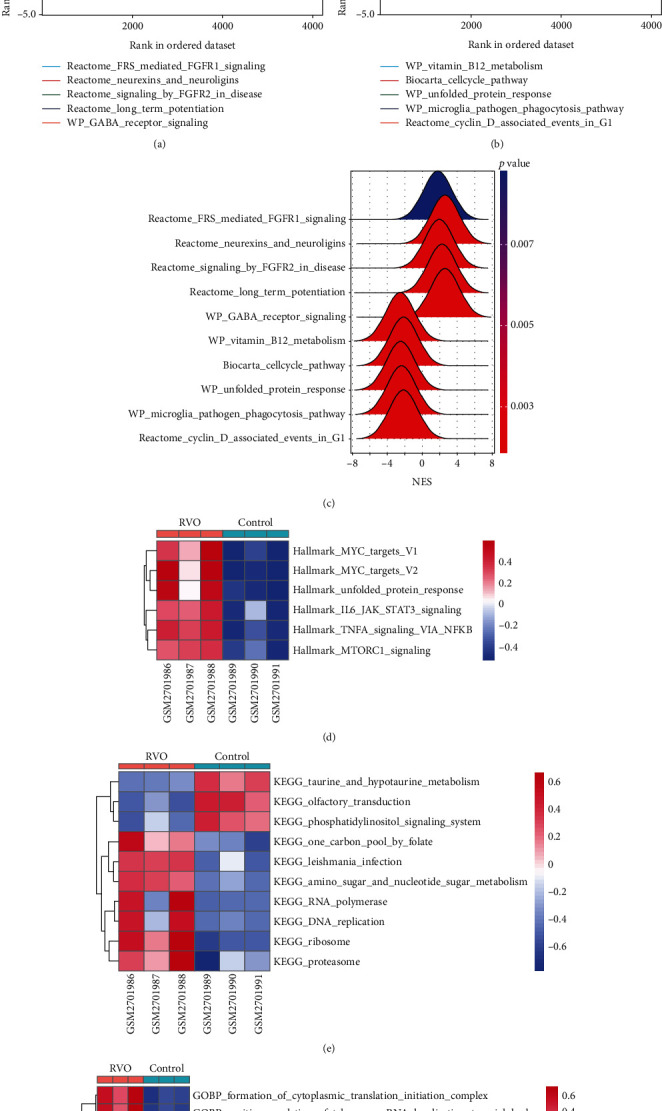
Gene set enrichment analysis (GSEA) and gene set variation analysis (GSVA) in GSE101398 dataset. (a, b) Multi-GSEA plot showed five most significantly upregulated pathways (a) and five most significantly downregulated pathways (b) in GSE101398 dataset. (c) Ridge plot displayed the normalized enrichment score (NES) of the above ten pathways. (d)–(f) Heatmap of GSVA result showed GSVA scores of the GO gene set (d), KEGG gene set (e), and hallmark gene set (f) enriched in GSE101398.

To further determine the possible molecules or pathways involved in RVO, GSEA was applied to compare RVO and normal retinal samples. Ten significant pathways, including 5 upregulated and 5 downregulated, were displayed in Figures 4(a)–4(c). Fibroblast growth factor receptor- (FGFR-) 1 signaling pathway, FGFR-2 signaling pathway, and gamma-aminobutyric acid (GABA) receptor signaling, which previously reported to be associated with inflammatory diseases [21–24], were demonstrated to be significantly upregulated in the RVO group, indicating that RVO was closely related with the status of inflammation.

GSVA was conducted to evaluate enrichment of gene set across samples using a nonparametric strategy. The hallmark gene sets, KEGG gene sets and GO gene sets including GO_BP, GO_CC, and GO_MF subsets were served as references. As shown in Figures 4(d) and 4(e), GSVA revealed that, in the RVO model, the significantly upregulated pathways were related with inflammatory events, such as TNF-*α*-NF-*κ*B signaling, interleukin 6 (IL6) signaling, and mechanistic target of rapamycin kinase complex1 (mTORC1) [25] signaling, which were consistent with GSEA results.

### 3.5. PPI Network Analysis

**Figure 5:**
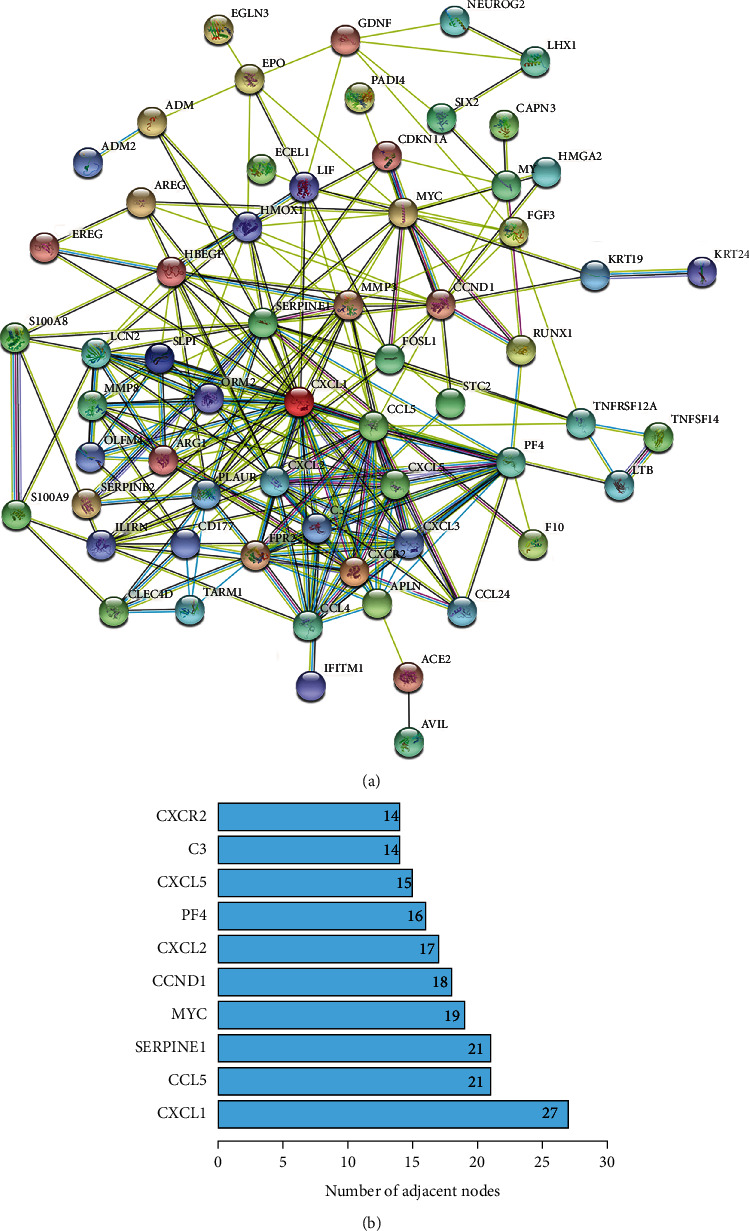
Protein-protein interaction (PPI) network. (a) The PPI network, consisting of 62 nodes and 255 edges in the network, was constructed using the STRING database. (b) Ten node genes with the most edges in the PPI network. STRING: Search Tool for the Retrieval of Interacting Genes; CXCL1/2/5: C-X-C motif chemokine ligand 1/2/5; CCL5: C-C motif chemokine ligand 5; SERPINE1: serpin family E member 1; MYC: MYC proto-oncogene; BHLH: transcription factor; CCND1: cyclin D1; PF4: platelet factor 4; C3: complement C3; CXCR2: C-X-C motif chemokine receptor 2.

The PPI network of DEGs was constructed based on STRING database and was shown as a cluster composed of 62 nodes and 255 edges (Figure 5(a)). Ten node genes with the most edges in the network were explored and displayed in Figure 5(b). Majority of these genes played crucial role in the inflammatory response.

### 3.6. Clinical Characteristic

The present study enrolled 11 eyes of 11 patients with RVO-ME, including 4 cases of CRVO and 7 cases of BRVO. Among these 11 eyes, 3 (27.27%) eyes had HRF in one slab, 2 (18.18%) eyes had HRF in two slabs, and 6 (54.55%) eyes had HRF in all three slabs (superficial slab, deep slab, and outer retina slab). The baseline clinical characteristics of 11 eyes were summarized in Table 1. A significant improvement of BCVA was observed after intravitreal injection of dexamethasone intravitreal implant, i.e., 0.63 ± 0.29 (logMAR, baseline) vs. 0.36 ± 0.17 (logMAR, postinjection) (*p* = 0.03). IOP of all patients was within the normal range, and no adverse events occurred during the follow-up.

### 3.7. Baseline BCVA Was Positively Correlated with SRF and Macular Thickness

As shown in Table 2, Pearson correlation revealed that BCVA at baseline was positively correlated with SRF area, retinal thickness in fovea, temporal of parafovea and perifovea, nasal of parafovea and perifovea, and inferior of parafovea. However, no correlation was detected between baseline BCVA and HRF number, as well as between BCVA and cystoid edema area.

### 3.8. Baseline HRF Number Was Negatively Correlated with Foveal Thickness

As shown in Table 3, HRF number at baseline was negatively correlated with foveal thickness, whereas no correlation was found between HRF number and other regions of macula. Moreover, the area of cystoid edema and SRF was not associated with baseline HRF number.

### 3.9. Intravitreal Injection of Dexamethasone Intravitreal Implant Was Effective to Decrease HRF Number, Cystoid Edema, and SRF Area

**Figure 6:**
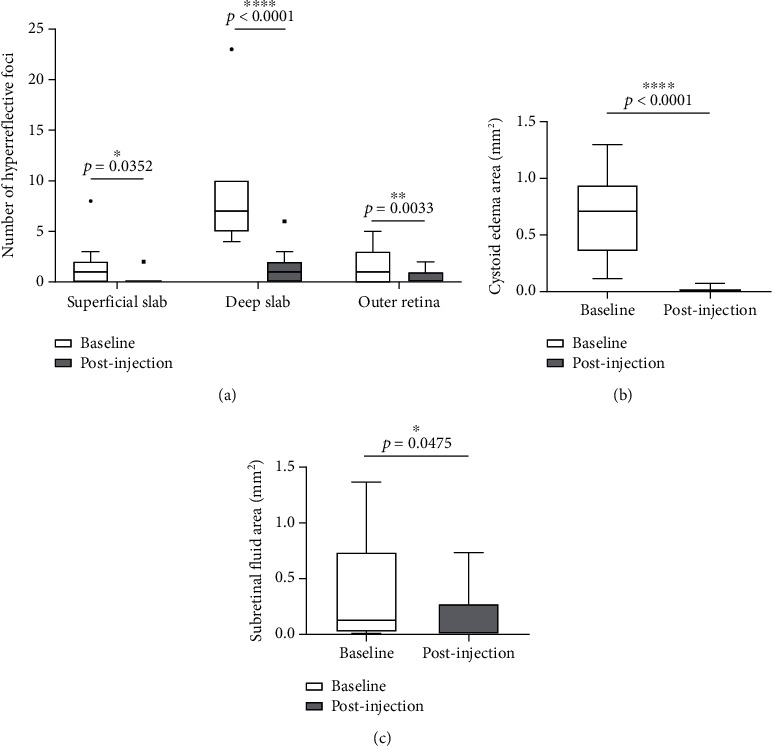
Dexamethasone intravitreal implant was effective for patients with RVO-ME. The changes of HRF number (a), cystoid edema area (b), and SRF area (c) were compared between the baseline and postinjection.

**Figure 7:**
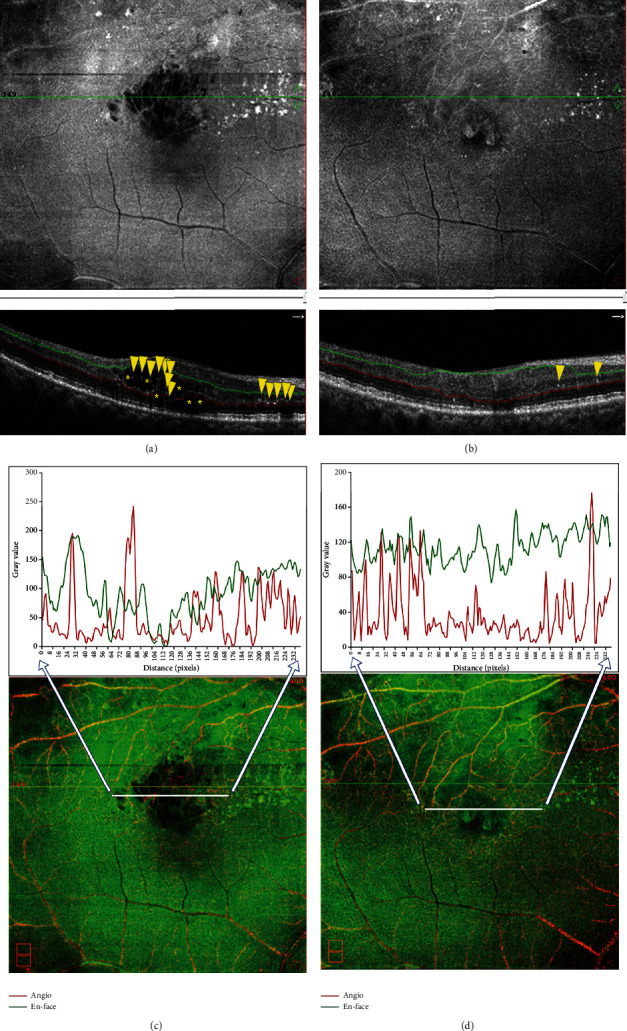
Representative images of ME secondary to branch retinal vein occlusion (BRVO-ME) before and after the intravitreal injection of dexamethasone intravitreal implant. (a) A 62-year-old female patient with BRVO-ME. Typical cystoid edema was observed in both En-face image and B-scan image of OCTA. HRF (yellow arrowhead) and cystoid edema (yellow asterisk) were pointed out in B-scan image of OCTA. (b) En-face and B-scan images showed remarkable alleviation of cystoid edema and reduction of HRF (yellow arrowhead) after treatment. (c, d) Merged images of both angiograph and En-face image (lower) at baseline (c) and post-injection (d). The gray value of the area crossed by horizontal lines, generated by ImageJ software, was displayed (upper). HRF: hyperreflective foci.

With OCTA, the changes of HRF number, cystoid edema area, and SRF area were analyzed and compared in patients with RVO-ME between baseline and postinjection. As shown in Figure 6(a), the number of HRF was significantly reduced after intravitreal injection of dexamethasone intravitreal implant. The representative images of HRF were illustrated in Figure 7. Furthermore, area of cystoid edema was significant decreased from 0.67 ± 0.33 mm^2^ (baseline) to 0.01 ± 0.02 mm^2^ (post-injection) (Figure 6(b)). Additionally, a significant reduction of SRF area was found after treatment (Figure 6(c)).

### 3.10. Retinal Vessel Density and Retinal Thickness Were Altered after Dexamethasone Intravitreal Implant Treatment

**Figure 8:**
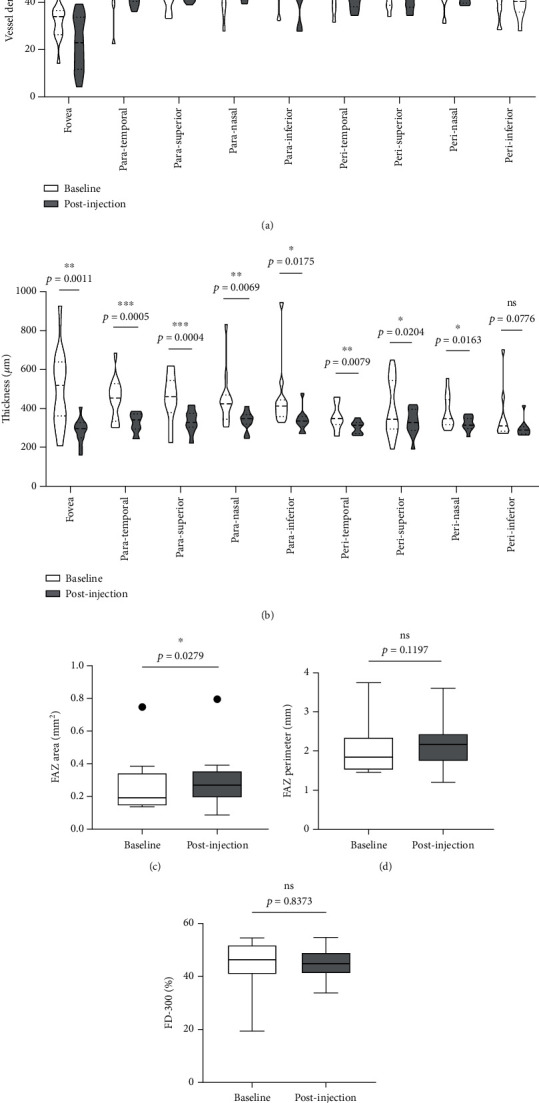
Comparison of vessel density and other related parameters. (a, b) Changes in retinal vessel density (a) and retinal thickness (b) at different regions of macula between baseline and post-injection. (c)-(e) Comparison of FAZ area (c), FAZ perimeter (d), and FD-300 (e) between baseline and post-injection. ns: not significant; FAZ: foveal avascular zone; FD-300: foveal vessel density within a 300 *μ*m wide region of FAZ.

We further analyzed retinal vessel density, retinal thickness, FAZ area, FAZ perimeter, and foveal vessel density within a 300 *μ*m wide region of FAZ (FD-300). Except for fovea and superior of perifovea area, no significant differences in vessel density were found in other regions before and after treatment (Figure 8(a)). Moreover, retinal thickness was significantly decreased in majority of macular regions apart from inferior of perifovea area (Figure 8(b)). A statistical significant increase of the FAZ area (Figure 8(c)), whereas no significant changes in FAZ perimeter (Figure 8(d)) and FD-300 (Figure 8(e)) were detected.

## 4. Discussion

RVO, with an estimated prevalence of 16.4 million globally [1], is the second most common vision-threatening retinal vascular disease after diabetic retinopathy, since there is no effective treatment to unblock the occluded retinal veins. Therefore, current treatments, aiming its complications such as ME and neovascularization, are of the great importance. Targeting VEGF, a vasopermeability cytokine, is widely used as the first-line therapy for the treatment of RVO. However, monthly intravitreal injection of anti-VEGF agent seemed less effective over time in a minority of patients, suggesting the involvement of other factors, e.g., inflammation-related factors, in the pathogenesis of RVO. Thus, the role of inflammation involved in RVO has become a subject of growing interest. Dexamethasone intravitreal implant presents beneficial outcome in the treatment of RVO-ME; however, corticosteroid-induced glaucoma and complicated cataract cannot be ignored [26]. Therefore, identification of other potential therapeutic targets would benefit the patient with RVO.

Due to less opportunities to obtain retinal samples from RVO patients for high throughput sequencing, animal model mimicking the clinical characteristics of RVO seems more practical. For example, laser photocoagulation with eosin Y or rose bengal as photosensitizer is used to establish the animal model [7, 27]. In our study, integrated bioinformatic analysis showed differential genes enriched in various inflammatory events in the gene expression profiling of the laser-induced mice RVO model (GSE101398). Interestingly, both GSVA and KEGG analysis found that TNF-*α* signaling, a canonical proinflammatory paracrine and endocrine mediator, was a significant pathway in RVO. Kachi et al. reported a case that foveal thickness of one patient with RVO-ME was decreased by 53.42% after 8 intravenous injections of infliximab, a TNF-*α* antibody, while the macular edema relapsed after discontinuation of infliximab, indicating TNF-*α*, as an inflammatory factor, that was involved in the pathogenesis of RVO-ME [28]. In addition, TNF-*α* is proved to be the upstream regulator of VEGF in the signaling cascade [29, 30] and lied in the crosstalk of angiogenesis and inflammation, suggesting specific blockade of TNF-*α* would be a promising alternative.

Besides TNF-*α*, C-X-C motif chemokine ligand 1 (CXCL1) that possess the most edges in the PPI network, might be another potential target. CXCL1 is a chemokine secreted by both immune and nonimmune cells [31]. CXCL1 promotes inflammatory response through interaction with the C-X-C motif chemokine receptor (CXCR)1 and CXCR2 receptors [32]. CXCL1 is also reported to exert angiogenesis effect via regulating the VEGF-A expression [33]. Collectively bioinformatic analysis offered targeting retinal inflammation as a prospective treatment for RVO.

HRF, initially described in diabetic macular edema (DME) patients, display as highly reflective dots on spectral domain-OCT images [17]. Previous publications hypothesized that HRF may correspond to activated microglia [34–36] and proposed HRF as a biomarker of inflammation in patients with age-related macular degeneration [37] and DME [38]. The studies on the correlation between baseline BCVA and HRF number showed controversial result. Lai et al. [39] and Segal et al. [40] found that the patients with poor BCVA have more HRF, while others failed to get this conclusion [36, 41–43]. In our study, no correlation was found between baseline BCVA and HRF via Pearson correlation analysis, but the amount of HRF was significantly decreased and BCVA improved after receiving intravitreal injection of dexamethasone intravitreal implant, suggesting the anti-inflammatory effect of dexamethasone to deactivate microglia.

As mentioned before, ME is the outcome of retinal fluid accumulation surrounding the fovea [44]. Fluid accumulation could be in the form of extracellular (resulting in the formation of cystoid edema space), intracellular (resulting in the swelling of Müller cells), or subretinal (resulting in the buildup in the subretinal space). Intracellular edema of Müller cells, with alteration of cellular ionic dispensation, forms cystoid space in OCTA images and contributes significantly to ME. Wang et al. [45] found ranibizumab could mitigate Müller cell intracellular edema under diabetic condition via the upregulation of K^+^ channel 4.1 (Kir4.1) and aquaporin 4 by binding with VEGF-A. Corticosteroids are applied to alleviate both extracellular and intracellular edema and therefore improve the clinical outcome after acute ischemic stroke [46]. In the present study, a significant reduction of cystoid space was observed after dexamethasone treatment, indicating that dexamethasone intravitreal implant might be beneficial in alleviating Müller glial intracellular edema.

**Figure 9:**
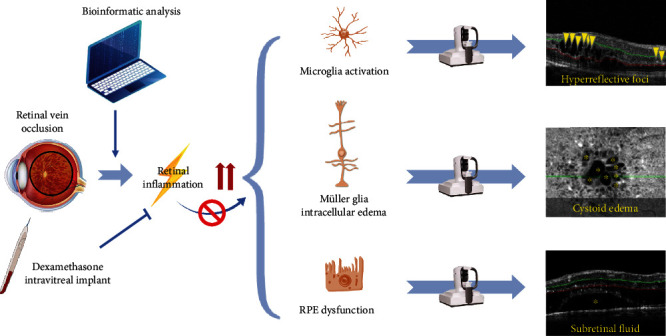
Schematic diagram depicts the involvement of retinal inflammation in RVO-ME, and dexamethasone intravitreal implant was effective to alleviate inflammation and improve clinical outcomes. HRF (yellow arrowhead), cystoid edema, and subretinal fluid (yellow asterisk).

Subretinal fluid accumulation often associates with RPE dysfunction and outer BRB breakdown. The underlying mechanism might be that the inflammation-related cytokines and factors, such as TNF-*α* and NF-*κ*B, were upregulated, resulting in the inflammatory responses and dysfunction of RPE [47]. In this study, a significant depletion of subretinal fluid was detected after intravitreal injection of dexamethasone intravitreal implant, indicating that dexamethasone might be helpful to preserving RPE function. Taken together, we hypothesized that retinal inflammation is upregulated in RVO, leading to microglia activation (HRF), Müller glial intracellular edema (cystoid edema), and RPE dysfunction (SRF) as evidence by OCTA. Dexamethasone intravitreal implant could reverse these detrimental effects of retinal inflammation (Figure 9).

There are some limitations in the current study. First, the proposed two targets: TNF-*α* and CXCL5 were analyzed based on the public database and lack of validation on the animal model. Second, the sample size of clinical study was relatively small, that may affect the comparison results. Last, the cystoid edema space is three-dimensional. But due to the methodology issue, we only quantified the area at the largest section of B-scan image.

## 5. Conclusions

Retinal inflammation served a crucial role in RVO pathogenesis, causing BRB breakdown, microglia activation, Müller glial intracellular edema, and RPE dysfunction, which could be assessed in OCTA as the imaging biomarkers. And thus, anti-inflammation treatment is suggested to be initiated soon after the diagnosis of RVO-ME, and OCTA characteristics could serve as the therapeutic biomarkers.

## Figures and Tables

**Table 1:** The baseline characteristics of 11 patients classified according to HRF distribution in different slabs.

The number of slabs with HRF	1	2	3
Eyes	3	2	6
Gender (male/female)	2/1	1/1	4/2
Age (years)	71.33 ± 2.36	61.50 ± 0.50	72.83 ± 7.71
Central macular thickness	635.70 ± 229.61	597.00 ± 41.00	404.00 ± 148.08
Visual acuity (logMAR)	0.83 ± 0.34	0.48 ± 0.23	0.58 ± 0.18
Subretinal fluid	2 (66.67%)	2 (100.00%)	4 (66.67%)
External limiting membrane disruption	2 (66.67%)	2 (100.00%)	4 (66.67%)
Ellipsoid zone disruption	2 (66.67%)	2 (100.00%)	5 (83.33%)

**Table 2:** Correlation between baseline BCVA and baseline OCTA characteristics.

	Variables	Coefficient	*p* value
BCVA (logMAR)	HRF number	-0.1436	0.6736
	SRF area	0.8682	0.0005^∗^
	Cystoid edema area	-0.1142	0.7381
	Thickness of fovea	0.7100	0.0144^∗^
	Thickness of temporal parafovea	0.7905	0.0038^∗^
	Thickness of nasal parafovea	0.7144	0.0135^∗^
	Thickness of superior parafovea	0.4481	0.1669
	Thickness of inferior parafovea	0.6371	0.0350^∗^
	Thickness of temporal perifovea	0.6372	0.0350^∗^
	Thickness of nasal perifovea	0.6307	0.0375^∗^
	Thickness of superior perifovea	0.2054	0.5446
	Thickness of inferior perifovea	0.5672	0.0688

**Table 3:** Correlation between baseline HRF number and OCTA characteristics.

	Variables	Coefficient	*p* value
HRF number	Cystoid edema area	-0.4706	0.1441
	Subretinal fluid area	0.0600	0.8609
	Thickness of fovea	-0.6143	0.0444^∗^
	Thickness of temporal parafovea	-0.4609	0.1536
	Thickness of nasal parafovea	-0.4445	0.1708
	Thickness of superior parafovea	-0.1665	0.6246
	Thickness of inferior parafovea	-0.4518	0.1630
	Thickness of temporal perifovea	-0.3107	0.3525
	Thickness of nasal perifovea	-0.2221	0.5117
	Thickness of superior perifovea	-0.0025	0.9941
	Thickness of inferior perifovea	-0.4390	0.1768

## Data Availability

All relevant data have been presented in the manuscript. Requests for or questions about the data can be addressed to 13917311571@139.com or xdsun@sjtu.edu.cn.
